# Health Professionals’ Alcohol-Related Professional Practices and the Relationship between Their Personal Alcohol Attitudes and Behavior and Professional Practices: A Systematic Review

**DOI:** 10.3390/ijerph110100218

**Published:** 2013-12-23

**Authors:** Savita Bakhshi, Alison E. While

**Affiliations:** Florence Nightingale School of Nursing and Midwifery, King’s College London, James Clerk Maxwell Building, 57 Waterloo Road, London SE1 8WA, UK; E-Mail: alison.while@kcl.ac.uk

**Keywords:** alcohol, attitudes, behavior, consumption, practice, doctor, nurse, health professional, health promotion, systematic review

## Abstract

Health professionals’ personal health behaviors have been found to be associated with their practices with patients in areas such as smoking, physical activity and weight management, but little is known in relation to alcohol use. This review has two related strands and aims to: (1) examine health professionals’ alcohol-related health promotion practices; and (2) explore the relationship between health professionals’ personal alcohol attitudes and behaviors, and their professional alcohol-related health promotion practices. A comprehensive literature search of the Cochrane Library, MEDLINE, EMBASE, PsycINFO, CINAHL, British Nursing Index, Web of Science, Scopus and Science Direct (2007–2013) identified 26 studies that met the inclusion criteria for Strand 1, out of which six were analyzed for Strand 2. The findings indicate that health professionals use a range of methods to aid patients who are high-risk alcohol users. Positive associations were reported between health professionals’ alcohol-related health promotion activities and their personal attitudes towards alcohol (*n* = 2), and their personal alcohol use (*n* = 2). The findings have some important implications for professional education. Future research should focus on conducting well-designed studies with larger samples to enable us to draw firm conclusions and develop the evidence base.

## 1. Introduction

### 1.1. Why is Alcohol-Related Health Promotion Important?

The harmful use of alcohol results in approximately 2.5 million deaths globally each year, with nearly 4% of all deaths worldwide attributable to alcohol use across developed and developing nations [1]. Around 6.13 liters of pure alcohol per person (aged 15 years and older) was consumed worldwide in 2005 [1]. Alcohol use is the third largest risk factor for around 60 diseases and disabilities, including different cancers, cirrhosis of the liver, cardiovascular diseases and epilepsy [1,2]. Heavy drinkers are also at greater risk of conditions such as hypertension, gastrointestinal bleeding, sleep disorders and depression [3]. Additionally, alcohol use is also associated with poor social outcomes such as relationship breakdown, trauma, violence, child neglect and abuse and workplace absenteeism [1,4]. 

### 1.2. Alcohol Use in Health Professionals

A large body of evidence suggests that a significant proportion of health professionals have high rates of alcohol use [5,6,7,8,9,10,11,12], with consumption increasing over time [13]. In a review paper, Baldisseri [14] estimated that approximately 10%–15% of all health professionals have misused alcohol or drugs at some time during their careers; around 14% of doctors have an alcohol use disorder and 6%–8% have substance use disorders. A recent survey of 3,213 Canadian doctors found that on days when they drank alcohol 1.3% of male and 0.8% of female doctors consumed five alcoholic drinks or more a day during the past year, and 12% of male and 4% of female doctors had done so in the past month [15]. In another survey of 1,784 Swiss primary care doctors 66% of the doctors consumed alcohol with 30% being at risk of harmful drinking. In comparison to a small sample of the Swiss general population, the doctors were more likely to drink alcohol (96% *vs.* 78%), and twice more likely to be at risk drinkers (30% *vs.* 15%) [16]. Past research conducted across five countries has also found that alcohol abuse in health professionals is related to various factors such as age, gender, personality traits and working long hours [7,8,13,16,17,18] although measures of alcohol abuse varied across the studies. For example, male doctors drank more frequently, consumed higher levels of alcohol per occasion and at a more hazardous or harmful level than female doctors [10,13]. Recent findings derived from single center cohort studies conducted in the UK and USA also indicate concerns relating to future health professionals with nursing students reporting high levels of alcohol use during their training [19,20,21]. Work-related stress may account for unhealthy coping habits such as alcohol use, smoking and/or using drugs for relaxation purposes [10].

### 1.3. Health Professionals’ Personal Alcohol Attitudes and Behaviors

Health professionals are ideally positioned to promote and improve the health and well-being of individuals, families and communities [22]. They are able to reach large proportions of the population as they are often viewed as role models by their patients, and as such are expected to practice what they “preach” [10,15]. However, interactions with patients and the level of intervention and care provided to patients may be determined by a variety of factors, including the health professionals’ personal and professional attitudes, beliefs and experiences of alcohol, as well as their own personal alcohol use [10,13,23]. Personal health beliefs and the importance that an individual attaches to their behaviors may also influence the adoption of health-related behaviors. For example, negative attitudes towards substance users have been reported by different groups of health professionals, including viewing caring for such patients as unrewarding and unpleasant [24]. Health professionals’ own alcohol use may also play an important role in their interaction with their patients [12]. For example, Crothers and Dorrian [23] found that nurses who consumed alcohol were more likely to believe that the danger is in the alcohol, and not in the person, thereby establishing a positive rapport with their patients. Thus, these factors may shape and influence relationships between health professionals and their patients. 

### 1.4. Using Screening Tools to Intervene with Patients Who have Alcohol-Related Problems

Screening and brief interventions (BIs) enable health professionals to educate their patients about the risks associated with alcohol use. Brief advice and personalized counseling sessions lasting 5–10 min conducted by health professionals can help reduce alcohol use in high-risk drinkers [25]. Murray *et al.* [26] identified two groups of patients who may require alcohol-related interventions: (1) the at-risk drinker who consumes alcohol at hazardous levels; and (2) patients who meet the diagnostic criteria for alcohol abuse or alcohol dependence. Various tools and frameworks have been developed to aid health professionals across a range of health behaviors [27]. One such framework is the widely-used 5-As behavioral counseling framework that can be applied to address a range of behaviors and health conditions including substance use and smoking [27]. It comprises five components: Assess, Advise, Agree, Assist, and Arrange. If patients are found to be at risk, then clinicians are able to advise them to change their behavior, assess their interest in changing, assist them in their efforts, and arrange appropriate follow-up [28]. Descriptions of each component as applied to alcohol use can be seen in Table 1. 

**Table 1:** The 5-As behavioural counseling framework applied to alcohol use.

The 5-As	Description
*Assess*	Alcohol use with a brief screening tool followed by clinical assessment as needed
*Advise*	Patients to reduce alcohol use to moderate levels
*Agree*	On individual goals for reducing alcohol use or abstinence (if indicated)
*Assist*	Patients with acquiring the motivation, self-help skills, and support needed for behavior change
*Arrange*	Follow-up support and repeated counseling, including referring dependent drinkers for specialty treatment

Screening tools can be used as part of the clinical assessment to determine the extent to which a patient may have harmful alcohol use. The Alcohol Use Disorders Identification Test (AUDIT) [29] is a 10-item questionnaire yielding a possible score of 40. Scores below 8 indicate low harm, between 8–15 indicate a medium or hazardous alco­hol use, 16 or greater indicate an increased level of harmful use, and scores of 20 and above indicate a need for further investigation. The first three items (AUDIT-C) assess whether a patient’s condition suggests hazardous drinking. A score of ≥5 for men and ≥4 for women indicates hazardous alcohol use that requires a BI and the administration of a full AUDIT. 

BIs conducted by health professionals are a common method of advising patients with high or harmful levels of alcohol use, and can help to moderate alcohol use [30]. BIs provide information about the hazardous or harmful risks associated with alcohol use and allows health professionals to make appropriate suggestions to their patients. The aim is to increase awareness of the risks associated with high alcohol use and to provide patients with the tools that can increase their motivation to make appropriate lifestyle and behavior changes.

### 1.5. Review Aims

Health professionals’ personal health behaviors have been found to be significantly associated with their professional health promotion practices related to smoking cessation [31], physical activity [32], and weight management [33,34], but little is known in relation to alcohol use. Previous studies investigating alcohol use and misuse in health professionals have indicated that this is a significant problem that can impact upon health professionals’ daily practices involving patients [17]. It is, therefore, timely to explore the personal alcohol-related attitudes and alcohol use of health professionals so that their effect on their professional alcohol-related health promotion practices can be examined. Thus, this review has two related strands and aims to: (1) examine health professionals’ alcohol-related health promotion practices; and (2) explore the relationship between health professionals’ personal alcohol attitudes and behaviors, and their professional alcohol-related health promotion practices. 

## 2. Methods

### 2.1. Search Strategies

The following electronic databases were searched to locate relevant published studies from January 2007–August 2013: the Cochrane Library, MEDLINE, EMBASE, PsycINFO, CINAHL, British Nursing Index, Web of Science, Scopus and Science Direct. Keyword combinations and specific search terms used can be found in Table 2. The search strategy involved using Boolean operators to combine the main terms such as (“alcohol” OR “substance”) AND (“doctor” OR “nurse”) AND (“attitude” OR “behavior”) AND (“practice” OR “promotion”), with variations as required for each database. Variations of the words “behavior” and “consumption” were used to search for papers reporting on health professionals’ personal alcohol use. The word “use” was not used due to the potentially high number of irrelevant records that such a search would have identified. The search was limited to the English language papers only. In addition, citations in eligible papers and previous reviews in the subject areas were examined for additional papers that met the inclusion criteria for the present review. 

**Table 2:** Search terms.

Facets	Search terms
Alcohol	Alcohol; substance; drink
Health professional	Health professional; healthcare professional; healthcare provider; medical professional; medical staff; doctor; physician; nurse
Attitudes and behavior	Attitude; belief; perception; view; behavior; consumption
Practices	Practice; health promotion; prevention; health education; intervention; healthcare delivery; counseling; advice

### 2.2. Eligibility Criteria

Papers were selected for inclusion in this review if they met the following criteria: (1) reported health professionals’ personal attitudes towards alcohol using validated scales, (2) reported health professionals’ personal alcohol use, (3) reported health professionals’ alcohol-related health promotion practices, (4) examined the relationship between health professionals’ personal attitudes towards alcohol or their personal alcohol use and their alcohol-related health promotion practices, (5) primary research, (6) paper was published in English, and (7) published between 2007–2013. Papers were not restricted by study design and were excluded from both strands of the review if the data from different health professionals were not reported independently. Unpublished studies and other grey literature were also excluded. 

### 2.3. Study Identification

Initial screening of papers was undertaken by the first author (SB), who identified potential papers meeting the inclusion criteria from the abstracts, which was then checked independently by the second author (AEW). Full texts were obtained for the relevant papers, and for those which did not include an abstract or it was unclear from the abstract whether the paper was relevant to the review. Both authors (SB and AEW) independently examined the full text of each paper to ensure that it met all the inclusion criteria. Any uncertainties were resolved by discussion.

**Figure 1:**
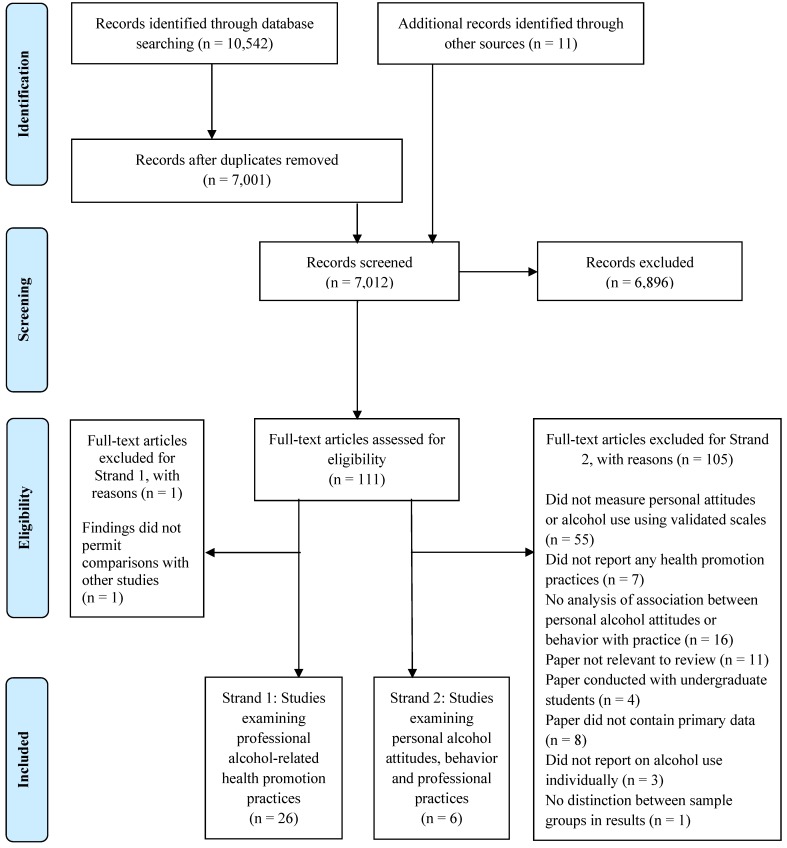
PRISMA flow chart.

A total of 117 full-text papers were assessed for eligibility for both strands of the review. Out of these, 27 were examined for the first strand focusing on professional alcohol-related health promotion practices. One paper was excluded [35] because only mean scores were reported. Thus, 26 studies were included in the first strand of the review. Six papers met the inclusion criteria for the second strand examining the relationship between health professionals’ personal alcohol attitudes and behaviors, and their professional alcohol-related health promotion practices. A summary of the literature identified at each stage of the search process can be found in the PRISMA flow chart [36] (Figure 1).

### 2.4. Data Extraction and Analysis

The first author (SB) extracted the following data from each included paper: country of study, study design and setting, study sample, measurements and main results. The second author (AEW) independently verified the extracted data, and made amendments as required. Both researchers conducted a joint quality appraisal, and came to an agreement about the quality of each paper using the Strobe checklist [37] and the CONSORT 2010 statement [38].

The 26 studies reporting professional alcohol-related health promotion practices (Strand 1) were categorized using the 5-As behavioral counseling framework [27], namely: Assess, Advise, Agree, Assist and Arrange. To permit comparisons between the studies, response options of “occasionally”, “some of the time”, “infrequently”, “seldom”, “rarely”, “never” and “no” were categorized as <50% of health professionals’ patients covered, whereas any response options of “always”, “all of the time”, “often”, “frequently”, “most of the time”, “as indicated”, “usually” and “yes” were categorized as >50% of patients covered. The baseline data from the randomized controlled trials (RCTs) and quasi-experiments and the latest data from the longitudinal studies were extracted alongside the cross-sectional survey data.

## 3. Results

### 3.1. Strand 1: Professional Alcohol-Related Health Promotion Practices

#### 3.1.1. Overview of Selected Papers

Twenty-six studies reported professional alcohol-related health promotion practices, out of which six were conducted in the UK, four in Australia, four in Sweden, two in the USA and the remaining 10 studies in other countries. The samples ranged from 50–4,946 participants and comprised doctors (*n* = 10), mixed samples of health professionals (*n* = 8), nurses (*n* = 2), midwives (*n* = 1) and other medical staff (*n* = 5). The studies were published in 2007 (*n* = 1), 2008 (*n* = 5), 2009 (*n* = 4), 2010 (*n* = 4), 2011 (*n* = 9) and 2012 (*n* = 3), respectively. 

Most of the studies employed cross-sectional surveys (*n* = 19), three were quasi-experiments, two were RCTs, and two were longitudinal studies designed to assess the effect of interventions upon professional activity. Half the studies (*n* = 13) recruited national samples of health professionals working in various settings, while the remainder were local and/or regional samples. The methodological quality of the studies was assessed using the criteria recommended by the EQUATOR Network for study designs [39]. Two studies were rated as high quality using the Strobe checklist [37], 10 studies were rated as moderate quality, and 12 were rated as weak quality due to various methodological limitations. The two RCTs [40,41] were assessed using the CONSORT 2010 statement [38] and were rated as moderate and high quality respectively. All of the studies employed researcher-developed instruments, and most (*n* = 21) collected data using a mail survey. A summary of each study is set out in Table 3.

#### 3.1.2. Professional Alcohol-Related Health Promotion Practices

The findings from the 26 studies reporting professional alcohol-related health promotion practices are presented in Table 4 and Table 5. Table 4 provides an overall summary of professional activity levels relating to alcohol-related health promotion, and Table 5 reports the effect of interventions upon professional activity. One study [42] did not specifically report the use of any components of the 5-As behavioral counseling framework [27], and is therefore not included in Table 4 or Table 5. The findings of this paper are discussed in the narrative synthesis.

**Table 3:** Professional alcohol-related health promotion practices: Summary of studies included.

Reference and location	Design and sample	Data collected	Quality rating
Aalto and Seppa, 2007, Finland [43]	Cross-sectional survey National census sample of doctors in primary health care centres *n* = 3,193 (61.0% response rate)	Researcher-developed questionnaire Views of when to advise patients about alcohol and their use of BIs	Moderate
Amaral-Sabadini *et al.*, 2010, Brazil [44]	Cross-sectional survey Random sample of staff in 5 primary health care centres in Sao Paulo *n* = 96 (60.0% response rate) (*n* = 30 doctors and nurses; *n* = 18 nursing assistants; *n* = 48 community health workers)	Researcher-developed questionnaire Clinical prevention practices, beliefs, satisfaction in working with people with alcohol use and readiness to implement BIs	Weak
Chun *et al.*, 2011, USA [45]	Cross-sectional survey Random sample of staff in academic pediatric emergency departments (ED) in Rhode Island *n* = 188 (21.2% response rate) (*n* = 81 doctors; *n* = 97 nurses; *n* = 10 doctors’ assistants)	Researcher-developed online questionnaire Beliefs about, attitudes towards, perceived barriers to and current practices related to adolescent patients drinking alcohol	Moderate
Demmert *et al.*, 2011, Germany [46]	Cross-sectional survey Census sample of gynaecologists in Schlewig- Holstein (Regional) *n* = 229 (64.0% response rate)	Researcher-developed questionnaire Attitudes towards BIs, assessment rates of alcohol use and obstacles to BIs	Weak
Fitzgerald *et al.*, 2009, UK [47]	Cross-sectional survey Census sample of pharmacists from 8community pharmacies in Greater Glasgow *n* = 222 (77.0% response rate)	Researcher-developed structured telephone interviews Current practice relating to patient alcohol use, views regarding role and training needs	Weak
Freeman *et al.*, 2011, Australia [48]	Cross-sectional survey National convenience sample of nurses in EDs *n* = 125 (40.0% response rate)	Researcher-developed questionnaire Assessment of patient alcohol use, advice and assistance regarding alcohol use	Moderate
Geirsson *et al.*, 2009, Sweden [49]	Cross-sectional survey Census sample of doctors in primary care in Skaraborg (Regional) *n* = 68 (52.0% response rate)	Researcher-developed questionnaire Perceptions of alcohol use among patients, advice and referral practices	Weak
Gross *et al.*, 2012, UK [50]	Cross-sectional survey National census sample of occupational health doctors in the National Health Service (NHS) across England, Scotland and Wales *n* = 145 (65.0% response rate)	Researcher-developed questionnaire Attitudes, practices and training needs regarding alcohol use	Weak
Holmqvist *et al.*, 2008, Sweden [51]	Cross-sectional survey National census sample of staff in primary health care *n* = 4,946 (52.0% response rate) (*n* = 1,821 doctors (47.0% response rate); *n* = 3,125 nurses (55.0% response rate))	Researcher-developed questionnaire Knowledge, attitudes and management of alcohol use	High
Holmqvist *et al.*, 2008, Sweden [52]	Cross-sectional survey National sample of occupational health staff in primary health care *n* = 1,072 (63.8% response rate) (*n* = 313 doctors (54.0% response rate); *n* = 759 nurses (69.0% response rate))	Researcher-developed questionnaire Assessment of patient alcohol use, current training and obstacles to BIs	Moderate
Indig, 2009, Australia [53]	Cross-sectional survey Convenience sample of ED doctors and nurses in two teaching hospitals in Sydney *n* = 78 (30.0% response rate) (*n* = 36 doctors; *n* = 42 nurses)]	Researcher-developed questionnaire Attitudes, beliefs, current practices and confidence levels regarding patient alcohol use	Weak
Kesmodel and Kesmodel, 2011, Denmark [54]	Longitudinal survey (9 year interval) Census sample of midwives in one antenatal care centre *n* = 51 (94.0% response rate)	Researcher-developed face-to-face structured interview Attitudes, knowledge and advice regarding alcohol use during pregnancy	Weak
Koopman *et al.*, 2008, South Africa [55]	Cross-sectional survey Random sample of doctors in private settings in Cape Town *n* = 50 (96.0% response rate)	Researcher-developed questionnaire Perceptions or assessment practices and obstacles to BIs	Weak
Lynagh *et al.*, 2010, Australia [56]	Cross-sectional survey Stratified random sample of ambulance officials in New South Wales (Regional) *n* = 264 (53.0% response rate)	Researcher-developed questionnaire Prevalence of accidents relating to patient alcohol use, and knowledge and practices regarding role	Weak
McCaig *et al.*, 2011, UK [57]	Cross-sectional survey National census sample of pharmacists in community pharmacies in Scotland *n* = 497 (45.0% response rate)	Researcher-developed questionnaire Views regarding patient alcohol use, knowledge, advice and practices	Moderate
Nilsen *et al.*, 2011, Sweden [58]	Quasi-experiment National census sample of occupational health staff in occupational and primary care *n* = 1,066 in 2005 (*n* = 309 doctors (53.0% response rate); *n* = 757 nurses (68.0% response rate)) *n* = 1,133 in 2008 (*n* = 331 doctors (61.0% response rate); *n* = 802 nurses (80.0% response rate))	Researcher-developed questionnaire Assessment of patient alcohol use, perceived knowledge and efficiency in practices and training needs	Moderate
Nygaard *et al.*, 2010, Norway [59]	Cross-sectional survey National random sample of doctors in primary health care *n* = 901 (45.0% response rate)	Researcher-developed questionnaire Attitudes towards and obstacles relating to assessment and BI practices regarding alcohol use	Moderate
Payne *et al.*, 2010, Australia [60]	Quasi-experiment Census sample of paediatricians in Western Australia (Regional) *n* = 82 (61.7% response rate)	Researcher-developed questionnaire Attitudes, knowledge and practices regarding alcohol use during pregnancy	Weak
Raistrick *et al.*, 2008, UK [61]	Cross-sectional survey Census sample of staff in six health authorities in Yorkshire and Humberside (Regional) *n* = 1,141 (42.0% response rate) (*n* = 100 doctors; *n* = 788 nurses; *n* = 228 health care assistants; *n* = 25 other medical staff)	Researcher-developed questionnaire Attitudes towards and assessment of patients and colleagues with substance misuse problems	Moderate
Seppanen *et al.*, 2012, Finland [62]	Quasi-experiment National census sample of doctors in primary health care centres *n* = 1,610 (50.9% response rate)	Researcher-developed questionnaire BI practices relating to patient alcohol use	Weak
Shepherd *et al.*, 2011, UK [63]	Cross-sectional survey National random sample of dentists in Scotland *n* = 175 (60.0% response rate)	Researcher-developed questionnaire Attitudes, subjective norms, perceived behavioural control, self-efficacy, knowledge, personal behaviour and intentions regarding alcohol-related practices	Weak
Tsai *et al.*, 2011, Taiwan [40]	Randomized controlled trial National random sample of nurses in 2 medical centres and 4 regional hospitals *n* = 395 (response rate not reported)	Researcher-developed questionnaire Knowledge, self-efficacy and clinical practice regarding patient alcohol use	Moderate
Vadlamudi *et al.*, 2008, USA [42]	Quasi-experiment Convenience sample of graduate nursing students at a single university *n* = 181 (response rate not reported)	Researcher-developed questionnaire Knowledge, attitudes and confidence in assessment and BI practices regarding patient alcohol use	Weak
van Beurden *et al.*, 2012, The Netherlands [41]	Randomized controlled trial National sample of doctors from 77 general practices *n* = 119 (2.8% response rate)	Researcher-developed questionnaire Assessment of patient alcohol use and advice-giving practices relating to alcohol	High
Vederhus *et al.*, 2009, Norway [64]	Cross-sectional survey Census sample of addiction staff in five southern counties of Health Region South East (Regional) *n* = 291 (79.7% response rate)	Researcher-developed questionnaires Attitudes and knowledge about the Twelve Step based self-help groups (TSGs) and current referral practices regarding alcohol use	High
Wilson *et al.*, 2011, UK [65]	Longitudinal survey (10 year interval) Random sample of doctors in 6 Primary Care Trusts (Regional) *n* = 282 (73.0% response rate)	Researcher-developed questionnaire Attitudes, practices and perceived facilitators and obstacles to BIs	Moderate

**Table 4:** A summary of the 5-As used in alcohol health promotion: Professional activity levels.

	Coverage of patients (>50%)						
	ASSESS		ADVISE		AGREE	ASSIST	ARRANGE
Study/5-A Element	Assess general ^a^ (%)	Assess using a screening tool ^b^(%)	Advise general ^c^ (%)	Advise specific ^d^ (%)	Agree general ^e^ (%)	Assist general ^f^ (%)	Arrange referral ^g^ (%)
Aalto and Seppa, 2007 [43]	−	−	−	19.6 Drs	60.4 Drs	−	−
Amaral-Sabadini *et al*., 2010 [44]	−	6.2	−	−	28.0	−	−
Chun *et al.*, 2011 [45]	10.4 RNs	−	−	24.2 RNs	−	−	18.4 RNs
Demmert *et al.*, 2011 [46]	33.6 Drs	−	−	−	35.0 Drs	−	36.0 Drs
Fitzgerald *et al.*, 2009 [47]	Not quantified Pharm	−	−	−	−	−	−
Freeman *et al.*, 2011 [48]	52.0 RNs	−	58.5 RNs	79.0 RNs	−	41.0 RNs ^j^	21.0 RNs ^k^
Geirsson *et al.*, 2009 [49]	−	−	−	12.0 Drs ^h^ 64.0 Drs ^i^	88.0 Drs ^h^ 36.0 Drs ^i^	−	90.0 Drs ^h^ 63.0 Drs ^i^
Gross *et al.*, 2012 [50]	28.0 MS	35.0 MS	−	−	−	−	59.0 Drs
Holmqvist *et al.*, 2008 [51]	28.0 RNs 50.0 Drs	−	−	−	−	−	−
Holmqvist *et al.*, 2008 [52]	85.0 RNs 70.0 Drs	80.0 RNs 50.0 Drs	−	−	−	−	−
Indig, 2009 [53]	91.7 Drsv45.2 RNs	5.7 Drs 4.9 RNs	−	−	17.7 Drs 14.6 RNs	25.7 Drs 42.9 RNs	28.6 Drs 26.2 RNs
Kesmodel and Kesmodel, 2011 [54]^l^	−	−	−	61.0 RMs	−	−	−
Koopman *et al.*, 2008 [55]	86.0 Drs	−	82.0 Drs	76.0 Drs	−	−	−
Lynagh *et al.*, 2010 [56]	40.0 MS	1.0 MS	−	4.0 MS	−	−	4.0 MS ^m^
McCaig *et al.*, 2011 [57]	18.9 Pharm	−	15.5 Pharm	18.9 Pharm	−	2.0 Pharm	−
Nygaard *et al.*, 2010 [59]	−	5.5 Drs	−	84.0 Drs	67.5 Drs	−	50.3 Drs
Raistrick *et al.*, 2008 [61]	40.0 MS	−	−	−	−	−	−
Shepherd *et al.*, 2011 [63]	−	−	−	17.0 Dents	−	−	−
Vederhus *et al.*, 2009 [64]	−	−	−	−	−	−	38.4 MS
Wilson *et al.*, 2011 [65]^ l^	40.0 Drs	−	−	−	−	−	−

Notes: **^a^** Informal discussions about alcohol use(*i.e.*, asking about quantity, frequency and alcohol use histories); **^b^** The use of one or more clinical assessment screening tools (*i.e.*, AUDIT [29]); **^c^** Advising or discussing about alcohol use relating to general lifestyle; **^d^** Advising or discussing about reducing alcohol use; **^e^** Discussing or advising on individual goals for reduction in alcohol use, and may include BIs for at risk drinkers; **^f^** Assisting with written information, goal setting, counseling and specialist support if needed; **^g^** Referring patients with alcohol problems to appropriate drug and alcohol counseling services; **^h^** These figures refer to dependent drinkers; **^i^** These figures refer to excessive drinkers; **^j^** Mean % across 4 items relating to assisting; **^k^** Mean % across 6 items relating to referral to various alcohol service; **^l^** Most recent set of findings are reported for these longitudinal studies; **^m^** 95% of staff also documented assessment, intervention and/or referral on the Patient Health Care Record; Drs = Doctors; RNs = Registered Nurses; Pharm = Pharmacists; MS = Mixed staff; Dents = Dentists.

**Table 5:** A summary of the 5-As * used in alcohol health promotion: The effect of interventions upon professional activity. (* No data is reported regarding Assist and Arrange).

			Coverage of patients (>50%)				
			ASSESS		ADVISE		AGREE
Study/5-A Element	Intervention description	Time points	Assess general ^a^ (%)	Assess using a screening tool ^b^(%)	Advise general ^c^ (%)	Advise specific ^d^ (%)	Agree general ^e^ (%)
Nilsen *et al.*, 2011 [58]	Risk Drinking Project (training, seminars and information provision)	Baseline	−	80.0 RNs ^f^ 50.0 Drs ^f^	−	−	−
		3 years follow-up	−	92.0 RNs ^f^ 79.0 Drs ^f^	−	−	−
Payne *et al.*, 2010 [60]	Educational resources	Baseline	22.4 Dr s ^g^	−	5.3 Drs ^h^	50.1 Drs ^i^	88.9 Drs^ j^
		6 months follow-up	21.7 Drs ^g^	−	10.1 Drs ^h^	28.1 Drs ^i^	68.3 Drs ^j^
Seppanen *et al.*, 2012 [62]	The Finnish Alcohol Programme (2004-2007) (information and support provision)	Baseline	−	−	−	−	9.3 Drs ^k^
		5 years follow-up	−	−	−	−	17.2 Drs ^k^
Tsai *et al.*, 2011 [40]	Alcohol Training Program (information provision and discussions)	Baseline	62.8 RNs ^l^	−	−	−	−
		1 month follow-up	61.5 RNs^m^	−	−	−	−
		3 months follow-up	65.8 RNs^n^				
Van Beurden *et al.*, 2012 [41]	Professionals, organizational and patient-directed activities program	Baseline	−	4.0 Drs ^o^	−	1.5 Drs ^q^	−
		1 year follow-up	−	9.0 Drs ^p^	−	3.5 Drs ^r^	−

Notes:^**a**^ Informal discussions about alcohol use (*i.e.*, asking about quantity, frequency and alcohol use histories); **^b^** The use of one or more clinical assessment screening tools (*i.e.*, AUDIT [29]); **^c^** Advising or discussing about alcohol use relating to general lifestyle; **^d^** Advising or discussing about reducing alcohol use; **^e^** Discussing or advising on individual goals for reduction in alcohol use, and may include BIs for at risk drinkers; **^f^** p > 0.05; **^g^** Prevalence Rate Ratio 0.97, 95% Confidence Interval 0.53–1.79; **^h^** Prevalence Rate Ratio 1.93, 95% Confidence Interval 0.59–6.30; **^i^** Mean % across 4 items relating to advising, therefore Prevalence Rate Ratio and Confidence Interval not reported here; **^j^** Prevalence Rate Ratio 0.77, 95% Confidence Interval 0.65–0.91; **^k^** Statistical analysis not reported; **^l^** t = −0.36, df = 393, *p* = 0.71; **^m^** F = 0.01, df = 1, *p* = 0.91; **^n^** F = 6.2, df = 1, *p* = 0.01; **^o^** *p* = 0.05; **^p^** *p* = 0.60; **^q^** *p* = 0.78; **^r^** *p* = 0.57; Drs = Doctors; RNs = Registered Nurses; RMs = Midwives.

### Professional Activity Levels

Table 4 shows that more health professionals focused on the first component of the 5-As behavioral counseling framework [27], namely, Assess. This involved health professionals asking their patients about their alcohol use in general, including the quantity and frequency of alcohol use, and recording their alcohol use histories. Five studies specifically reported health professionals’ use of one or more clinical assessment screening tools, for example, AUDIT [29]. Between 10.4%–91.7% of health professionals reported more than 50.0% patient coverage when conducting general assessments, whereas between 1.0%–80.0% reported more than 50.0% coverage of their patients when assessing patients using screening tools.

Providing advice or discussing alcohol use in general was also a common practice across the studies (*n* = 10). Three studies examined advice relating to alcohol use and general lifestyle and 10 studies reported advice relating to alcohol use reduction. Across the studies between 4.0%–84.0% of the health professionals reported offering alcohol-related advice to more than 50% of their patients. The highest coverage for both general (82.0%) and specific advice (84.0%) was reported by doctors. 

Only six studies reported health professionals’ practices relating to discussing or advising on individual goals for the reduction in alcohol use, and BIs for at-risk drinkers. Between 14.6%–88.0% of health professionals reported discussing or advising on individual goals and BIs with over 50% of their patients, with doctors reporting the highest coverage (88.0%). In three studies between 2.0%–42.9% of the health professionals assisted over 50% of their patients with written information, goal setting, counseling and specialist support if needed, with Registered Nurses reporting the highest coverage (42.9%) amongst the health professionals examined. In nine studies between 4.0%–90.0% of the health professionals referred over 50% of their patients with alcohol problems to appropriate drug and alcohol counseling services with doctors reporting the highest referral rates (90.0%). 

Eleven studies reported the percentage of health professionals who responded with “never” or “no” (*i.e.*, 0% coverage of patients) for each of the 5-As [40,42,43,46,48,49,52,53,54,56,60]. Between 2.4%–29.0% of health professionals never assessed by asking their patients about their alcohol use and between 13.0%–98.0% had never used a screening tool. Between 1.6%–30.8% of health professionals never provided advice relating to alcohol use and general lifestyle, and between 19.7%–83.0% reported not providing advice relating to alcohol use reduction. Two studies [40,56] reported that 10.9%–39.6% health professionals’ had never discussed or advised on individual goals for the reduction in alcohol use and BIs for at-risk drinkers with their patients. Another study [54] reported that 60.8% of their sample of pharmacists never provided their patients with alcohol-related written information, goal setting, counseling and specialist support if needed. Finally, between 9.5%–82.0% of health professionals across four studies [42,46,53,56] reported that they never referred their patients to appropriate drug and alcohol counseling services.

### The Effect of Interventions upon Professional Activity

Table 5 reports the effect of five interventions upon professional activity. The interventions included a mixture of information and support provision, and educational activities or resources. Two studies investigated patient assessment of their alcohol use in general, whereas three studies examined the use of clinical assessment screening tools. Between 21.7%–65.8% of health professionals reported conducting general assessments with over 50.0% of their patients, and between 4.0%–92.0% reported using screening tools with more than 50.0% of their patients. One study reported a decrease in general assessment by doctors at follow-up [60], and another study reported an overall positive effect of the intervention on general assessment rates over the study period [40]. 

Only two studies examined the effect of an intervention upon advice giving. One study investigated specific advice relating alcohol use reduction [41] while the other examined both general and specific advice [60]. Between 5.3%–50.1% of health professionals reported advising over 50.0% of their patients. There was a reported increase in general advice to patients at 6-months follow-up (from 5.3%–10.1%), although the same study reported a decrease in specific advice for the same period (from 50.1%–28.1%) [60]. 

Two studies examined the health professionals’ reported practices relating to discussing or advising on individual goals for the reduction in alcohol use, and BIs for at-risk drinkers. Between 9.3%–88.9% of the health professionals reported discussing or advising on individual goals and BIs with over 50% of their patients. Payne *et al.* [60] reported a decrease in health professionals discussing or advising on individual goals and BIs with their patients over the six month study period (from 88.9% to 68.3%). While Seppanen *et al.* [62] reported an increase from 9.3% to 17.2% in the health professionals discussing or advising at the end of five years. 

Table 5 does not report data regarding the Assist and Arrange components as the five studies did not investigate these components of the 5-As behavioral counseling framework. 

An additional study [42] that did not specifically report the use of any components of the 5-As behavioral counseling framework [27], reported that there was a significant positive effect of an educational intervention on personal attitudes, beliefs and confidence levels relating to conducting alcohol-related health promotion practices. The intervention focused on topics such as the steps involved in screening and intervention techniques and how to attain the desired patient goal. Nurses with little, moderate or no past experience with alcohol showed greater improvement in confidence post-intervention in relation to their professional alcohol-related practices. 

Only one study reported the percentage of health professionals who responded with “never” or “no” (*i.e.*, 0% coverage of patients) for each of the 5-As. Seppanen *et al.* [62] reported that 40.8% of their sample of doctors had not offered BIs to their patients at baseline, and 21.5% had not offered BIs at follow-up. 

### 3.2. Strand 2: Relationships between Professional Alcohol-Related Health Promotion Practices, Personal Attitudes and/or Alcohol Use

#### 3.2.1. Overview of Selected Papers

Six studies reported relationships between professional alcohol-related health promotion practices, personal attitudes and/or alcohol use, out of which two were conducted in the UK, one in Australia, one in the USA, one in Finland and one in Sweden. The samples ranged from 68–3,193 participants and comprised doctors (*n* = 2), nurses (*n* = 2), dentists (*n* = 1), and a mixed sample of health professionals (*n* = 1). The studies were published in 2007 (*n* = 1), 2008 (*n* = 2), 2009 (*n* = 1) and 2011 (*n* = 2).

**Table 6:** Studies included in the review.

Reference and location	Design and sample	Instruments and data collected	Key findings	Quality rating
Aalto & Seppa, 2007,Finland [43]	Cross-sectional survey National census sample of doctors in primary health care centers *n* = 3,193 (61.0% response rate)	Researcher-developed questionnaire Personal alcohol use AUDIT [29] Professional practices Use of BIs Advice to patients Other Demographic information Professional background (*i.e.*, specialism licence, practice location, and length of experience)	Doctors’ personal alcohol use (*i.e.*, mean units consumed) not reported Patient alcohol use threshold for intervention: 14.8 drinks/week for males and 10.6 drinks/week for females Doctors with high AUDIT scores reported higher mean thresholds for patient alcohol use for both male (*p* < 0.001) and female (*p* < 0.001) patients Use of BI associated with advising at higher thresholds (weekly drinking) for male and female patients (*p* < 0.001) Considering patients’ opinions when making recommendations associated with advising at higher thresholds (weekly drinking) for male (*p* = 0.02) and female (*p* = 0.05) patients Higher personal use of alcohol, use of BI, experience and age explained 9.0% of the variance for advising male patients Higher personal use of alcohol and use of BI explained 8.0% of the variance for female patients	Moderate
Freeman *et al.*, 2011, Australia [48]	Cross-sectional survey National convenience sample of nurses in EDs *n* = 125 (40.0% response rate)	Researcher-developed questionnaire Personal alcohol use ◦ Alcohol use in the last 30 days Personal attitudes ◦ Regarding discussions with patients about their alcohol use ◦ Ranking of the five most important beliefs Professional practices ◦ Assessment practices ◦ Estimation of number of patients seen in last week and BIs implemented Other ◦ Demographic information ◦ Length of experience in ED ◦ Alcohol specific training undertaken ◦ Role adequacy and Role legitimacy subscales (Alcohol and Alcohol Problems Perception Questionnaire (AAPPQ)) [66,67] ◦ Role overload and Freedom subscales (Michigan Organization Assessment Questionnaire) [68] ◦ Co-worker support and Supervisor support subscales (Job Content Questionnaire) [69]	26.0% (95.0% CI 19%–34.0%) reported drinking alcohol at a high risk level at least once in the last 30 days Knowing how to ask about alcohol sensitively (35.0%) & having a good rapport with patients (12.0%) rated as most influencing beliefs on asking patients about their alcohol use Busyness of ED (11.0%) rated as most important for being able to assist patients Nurses asked 26.3% (IQR 6.7%–72.7%) of patients about their alcohol use 35.0% (IQR 2.7%–10.9%) had breathalysed at least one patient in the week preceding the survey Nurses more frequently advised patients regarding alcohol use (52.0%), than assist (41.0%) or arrange (21.0%) Although organizational policy, supervisor support, personal alcohol use, role legitimacy and adequacy predicted theoretical determinants of behavior, they did not predict self-reported professional practices	Moderate
Geirsson *et al.*, 2009, Sweden [49]	Cross-sectional survey Census sample of doctors in primary care in Skaraborg (Regional) *n* = 68 (52.0% response rate)	Researcher-developed questionnaire Personal alcohol use ◦ AUDIT-C [29] ▪ Frequency and quantity of drinking and binge drinking in a single occasion Professional practices ◦ Discussions of alcohol use ◦ Recording patients’ weekly alcohol use ◦ Providing advice about alcohol ◦ BI referral patterns Other ◦ Estimations of following using vignettes: ▪ Severity of the patients’ alcohol use ▪ Importance of abstinence ▪ Confidence in helping patient to alleviate their alcohol-related problems ◦ Demographic information	Doctors’ personal alcohol use (*i.e.*, mean units consumed) not reported Recommendations to cut down on drinking were more frequent for excessive male drinkers than females (83.0%*vs.* 47.0%, *p* = 0.003) No differences for recommendations for male (17.0%) and female (8.0%) dependent drinkers Advice to cut down and abstain completely was more frequent for females than males (*p* = 0.0025) Female drinkers were more likely to be referred to BIs than males (*p* = 0.03) Doctors with AUDIT-C score ≥ 3 advised at significantly higher thresholds for both male (146 g/week) (*p* = 0.0026) female (103 g/week) (*p* = 0.0091) patients, than doctors with a score of ≤ 2 (89 g/week and 68 g/week respectively) No association between the kind of advice provided and doctors’ personal alcohol use	Weak
Raistrick *et al.*, 2008, UK [61]	Cross-sectional survey Census sample of staff in six health authorities in Yorkshire and Humberside (Regional) *n* = 1,141 (42.0% response rate) (*n* = 100 doctors; *n* = 788 nurses; *n* = 228 healthcare assistants; *n* = 25 other medical staff)	Researcher-developed questionnaire combining existing questionnaires Personal alcohol use ◦ Questions on personal alcohol use Personal attitudes ◦ Modified Alcohol and Alcohol Problem Perceptions Questionnaire (AAPPQ) [66] Professional practices ◦ Action to take on colleagues’ problem use Other ◦ Demographic information ◦ Work-related problems of self and colleagues (Alcohol Problems Questionnaire) [70]	93.0% consumed alcohol; mean units consumed amongst drinkers in the last week = 10.8 units 22.0% doctors, 17.0% nurses & 9.0% drank >21 units for men & 14 units for women Personal alcohol use higher for nurses and health care assistants than doctors (*p* = 0.034) Men drank more than women (*p* < 0.001) 2.0% knew 5 or more colleague affected at work by substance use in the last month 40.0% knew 5 or more colleagues who openly spoke about their alcohol use Perceptions of colleagues’ problems were significantly different for the “users” (*i.e.*, over safer alcohol limits) than the “social” group (*p* < 0.001) Overall trends showed that ‘Users’ had more favourable attitudes towards helping patients with substance misuse problems than “socials”	Moderate
Shepherd *et al.*, 2011, UK [63]	Cross-sectional survey National random sample of dentists in Scotland *n* = 175 (60.0% response rate)	Researcher-developed questionnaire Personal alcohol use AUDIT [29] Personal attitudes ◦ Agreement on possible consequences and usefulness to providing alcohol-related advice to patients Professional practices ◦ Advice about alcohol use ◦ Intention to provide advice about alcohol Other ◦ Subjective norms ◦ Perceived behavioural control ◦ Self-efficacy ◦ Knowledge ◦ Demographic information	Doctors’ personal alcohol use (*i.e.*, mean units consumed) not reported 85.0% had an AUDIT score of ≤ 8; 14.0% moderate levels of harmful drinking; 1.0% bordered on dependence 83.0% had not provided advice about alcohol use in the past 10 working days Intention to provide advice was associated with attitudes (*p* < 0.01), perceived behavioural control (*p* < 0.01), subjective norms (*p* < 0.01) and self-efficacy (*p* < 0.01) Personal attitudes towards alcohol, subjective norms and self-efficacy explained 35.0% of the variance in the intention to provide advice Knowledge and personal alcohol use not significantly related to intention to provide advice	Weak
Vadlamudi *et al.*, 2008, USA [42]	Quasi-experiment Convenience sample of graduate nursing students at a single university *n* = 181 (response rate not reported)	Researcher-developed questionnaire Personal alcohol use ◦ Questions on own problem with alcohol Personal attitudes ◦ Attitudes and beliefs about alcohol abuse and treatment Professional practices ◦ Assessment of alcohol use ◦ Advice provision about alcohol abuse ◦ Negotiating a measureable goal providing follow-up support Other ◦ Confidence levels in assessment ◦ Past experience with patients who abused alcohol ◦ Knowing someone other than patients with alcohol problems ◦ Demographic information	Nurses’ personal alcohol use (*i.e.*, mean units consumed) not reported Nurses with little, moderate or no past experience with alcohol showed greater improvement in confidence post-intervention in relation to their professional alcohol-related practices (*p* value not reported) Significant positive effect of educational intervention on attitudes, beliefs and confidence levels (*p* < 0.01) No significant modifying effect of age, education, own problems with alcohol or knowing someone with alcohol problems Significant modifying effect of past experience with patients who abused alcohol (*p* = 0.032)	Weak

Most of the studies employed a cross-sectional survey (*n* = 5), and one was a quasi-experiment. Half the studies (*n* = 3) recruited national samples of health professionals working in various settings, while the remainder were local and/or regional samples. Two papers reported the health professionals’ personal alcohol use, and four studies reported both their personal attitudes and alcohol use at baseline. Three papers were rated as moderate quality, and three were rated as weak quality due to methodological limitations using the Strobe checklist [37]. All of the studies employed researcher-developed instruments, and most (*n* = 5) collected data using a mail survey. A summary of each study is set out in Table 6.

#### 3.2.2. Instruments Used to Measure Personal Attitudes and Alcohol Use

Although all of the study questionnaires were developed by the researchers, additional instruments were also used. Two studies used the Alcohol and Alcohol Problem Perceptions Questionnaire (AAPPQ) [66,67], which has six sub-scales comprising a series of statements about working with patients with alcohol-related problems. Respondents are asked to indicate the strength of their agreement on a 7-point Likert scale ranging from “strongly agree” to “strongly disagree”. Three studies used the AUDIT [29] to measure health professionals’ personal alcohol use. The psychometric properties of the researcher-developed measures used were not reported in any of the six papers.

#### 3.2.3. Association between Personal Attitudes towards Alcohol and Professional Alcohol-Related Health Promotion Practices

Four studies reported an association between health professionals’ personal attitudes towards alcohol and their professional alcohol-related health promotion practices [42,48,61,63]. All four studies assessed personal attitudes and beliefs using researcher-developed questionnaires with no reference to their psychometric properties. One study included the Alcohol Problems Questionnaire [70] as part of their questionnaire, and two studies conducted preliminary qualitative research (*i.e.*, structured telephone interviews and exploratory semi-structured interviews) to inform the development of their questionnaire items [48,63]. 

Two studies [42,63] reported a positive association between the health professionals’ personal attitudes towards alcohol and their professional alcohol-related health promotion practices. A small study of dentists (*n* = 175) found that personal attitudes were significantly associated with the intention to provide professional alcohol advice [63]. Regression analysis showed that the personal attitudes of the dentists explained 30.0% of the variance regarding the intention to provide professional alcohol-related advice, out of a total of 35.0% which also included self-efficacy and subjective norms. Another study [42] found that an educational intervention had a significant positive effect on nurses’ personal attitudes regarding alcohol abuse and its treatment. The intervention included four steps based on the 5-As behavioral counseling framework [27], including: raising the subject of alcohol with patients, assessing and screening for alcohol use and possibly abuse, advising the patients with appropriate feedback, and negotiating measurable goals with patients with follow-up and support for patients. While the nurses’ own alcohol use, knowing someone with an alcohol problem, age or educational level had no modifying effect upon personal attitudes, beliefs and confidence levels, past experience with patients who had abused alcohol was related to reported professional practices (*p* = 0.032) suggesting the importance of prior experience to the formation of attitudes related to professional practice. 

Freeman *et al.* [48] asked nurses to rank the importance of their professional alcohol-related practices. Knowing how to ask sensitively about alcohol use (67.0%), having a good rapport with the patient (66.0%) and having a non-judgmental view (66.0%) were ranked the most important when asking patients about their alcohol use. Busyness of the emergency department (74.0%), knowing how to help the patient manage their alcohol use (65.0%) and having a drug and alcohol unit or drug and alcohol nurses in the hospital (61.0%) were rated as the most important when assisting patients to manage their alcohol use. The remaining study [61] of a mixed sample of health professionals reported that overall personal attitudes scores were not related to their professional alcohol-related health promotion practices. 

#### 3.2.4. Association between Personal Alcohol Use and Professional Alcohol-Related Health Promotion Practices

Six studies explored health professionals’ personal alcohol use and their professional alcohol-related health promotion practices [42,43,48,49,61,63]. Two out of the six multivariate analyses showed that there was a positive association between health professionals’ personal alcohol use and their professional alcohol-related health promotion practices [43,49]. In a large study of doctors (*n* = 3,193), Aalto *et al.* [43] found a positive association between the doctors’ AUDIT scores and the advice that they gave their patients. Doctors with high AUDIT scores (*i.e.*, ≥8) advised at significantly higher thresholds of alcohol use for both their male and female patients. The thresholds for the mean number of drinks per week for male patients was 17.9 (SD = 6.9), and 8.0 (SD = 3.0) for female patients. The doctors’ AUDIT scores for both male (*r* = 0.074, *p* < 0.001) and female (*r* = 0.072, *p* < 0.001) patients collectively explained 7.0%–8.0% of the variance regarding the threshold level of alcohol use at which to advise patients to reduce their drinking levels. Similarly, Geirsson *et al.* [49] also found that doctors with AUDIT-C scores of ≥3 advised at significantly higher thresholds for both male (146 g/week) (*p* = 0.0026) and female (103 g/week) (*p* = 0.0091) patients, than doctors with scores of ≤2 (89 g/week and 68 g/week respectively). Thus, the doctors’ own personal alcohol use had a significant effect on their professional practices with their patients. However, there appeared to be no relationship between the kind of advice provided and the doctors’ alcohol use in these two studies. 

Another large study of mixed health professionals [61] (*n* = 1,141) reported that health professionals who frequently consumed alcohol were likely to report more work-related problems for themselves and their colleagues compared to those who did not drink alcohol (*p* < 0.001). Health professionals who frequently consumed alcohol knew more colleagues who were affected by substance use at work in the last month, and knew more colleagues who spoke openly about their alcohol drinking than health professionals who consumed alcohol less often (*p* < 0.001). The remaining three studies reported that personal alcohol use was not associated with self-reported professional alcohol-related practices [48] or the intention to provide professional alcohol-related advice [63], or nurses’ own problems with alcohol use and their professional practices [42]. It is unclear why there was no consistent relationship between personal alcohol use and professional alcohol-related health promotion practices, however, only two studies reported doctors’ personal alcohol use in terms of mean units consumed making it difficult to compare the findings across different studies and their relevance to other health professionals.

## 4. Discussion

### 4.1. Overview of Findings

This review aimed to: (1) examine health professionals’ professional alcohol-related health promotion practices; and (2) explore the relationship between health professionals’ personal alcohol attitudes and behaviors, and their professional alcohol-related health promotion practices. In summary, the findings from Strand 1 indicated that a range of professional alcohol-related health promotion practices are currently being conducted using the 5-As behavioral counseling framework [27]. However, the studies varied in terms of the data collection instruments used to assess professional alcohol-related health promotion practices, making it difficult to synthesize the results. There are a number of possible explanations for the range of professional alcohol-related health promotion activities. Health professionals may not use the 5-As behavioral counseling framework for a number of reasons, including: a lack of confidence [71], a lack of knowledge about alcohol use (*i.e.*, what constitutes a unit) and related risk factors [57,71], a lack of time [52,65,72] and/or a lack of training and/or uncertainty about if and how they should raise the topic with their patients [65,72]. These factors may act as barriers to providing appropriate assistance to patients [73].

Furthermore, we only identified six studies that examined the effect of interventions upon professional activity. This made it difficult to reach firm conclusions about how and why health professionals’ alcohol-related health promotion practices changed after participation in interventions. More detailed accounts of interventions that are successful in changing behavior are required so that their potential for widespread use can be assessed.

Two studies in Strand 2 reported a positive association between the health professionals’ personal alcohol use and their professional alcohol-related health promotion practices and another two studies reported a positive association between the health professionals’ personal attitudes towards alcohol and their professional practices. These findings tentatively indicate that the health professionals’ personal alcohol use and attitudes may play a role in their professional practices with their patients. However, we cannot be certain about the associations between these factors, unless we increase the number of rigorous studies examining these relationships. 

### 4.2. Methodological Quality of the Included Studies

Most of the studies were cross-sectional surveys (*n* = 19), and all collected self-reported data relating to personal alcohol-related attitudes, personal alcohol use and professional alcohol-related health promotion practices. Data collected using these methods are prone to measurement error which may undermine the validity of the findings. Variations in sampling, settings and measurement also prevented us from drawing firm conclusions from the data. For example, the samples varied in terms of health professionals included (*i.e.*, doctors, nurses, midwives, pharmacists, dentists and mixed staff), sampling techniques, sampling frames (national *n* = 13; regional/local *n* = 13) and sample size (50 [55] to 4,946 [51]). Additionally the response rates across the studies ranged from 2.8% [41] to 96.0% [55], with one study not reporting their response rates [40]. These variations raise questions about the generalizability of the findings to wider groups of health professionals within different countries. Furthermore, there were few commonalities between the studies in terms of the instruments used to assess the health professionals’ personal alcohol-related attitudes, personal alcohol use and professional alcohol-related health promotion practices, and the way in which alcohol use was reported. The exception was the AUDIT [29] which was used in three studies (in Strand 2) to assess the health professionals alcohol use but none of the six studies reported the psychometric properties for their measurement tools. It should also be noted that only two papers (out of 26) were rated as high quality indicating the absence of rigorous studies in this field of enquiry. Thus, our understanding of the factors associated with health professionals’ alcohol-related health promotion practices must remain limited. Future research examining health professionals’ alcohol-related health promotion practices and their related factors should comprise well-designed studies (*i.e.*, with prospective designs) including larger and representative samples using valid and reliable measures.

### 4.3. Strengths and Limitations of the Review

To our knowledge, this is the first review to investigate the relationship between health professionals’ personal attitudes and alcohol use, and their professional alcohol-related health promotion practices reported in published studies between 2007 and 2013. A wide range of search terms and their variations were adopted to retrieve all potential papers published in English using strict criteria to include only relevant studies. The review included different groups of health professionals, including: doctors, nurses, midwives, dentists, pharmacists, and other medical staff working in a variety of settings. The findings from Strand 1 indicated that a range of health professionals include alcohol-related health promotion activities with their daily clinical practices, however, the relationship between personal alcohol use and personal attitudes towards alcohol and the professional alcohol-related health promotion activities requires further investigation. 

A limitation of this review is that we found only six studies that investigated the relationship between the health professionals’ personal alcohol attitudes and behaviors, and their professional alcohol-related health promotion practices published between 2007 and 2013, which limits the strength of the empirical evidence and the conclusions that we are able to draw. The inclusion of qualitative studies that explore the relationship between personal attitudes, alcohol use and professional alcohol-related health promotion practices [74] and studies conducted in other languages in addition to English may have identified other relevant studies for inclusion in the review. Furthermore, limiting the review to published, peer-reviewed studies leaves the review open to the possibility of publication bias. Therefore, including grey literature in addition to published studies may have strengthened the review. Nevertheless, this review has highlighted the need for rigorous studies exploring the relationship between health professionals’ personal behavior and their professional practice. 

### 4.4. Implications for Practice and Future Research

This review has highlighted some important implications for clinical practice and future education and training programs for health professionals. Some studies have indicated a lack of knowledge about alcohol (*i.e.*, what constitutes a unit) [57,71] with a lack of confidence in raising alcohol-related concerns with patients [71] potentially determining the level of care that health professionals provide. Thus, it is important to identify and address potential barriers that may be preventing health professionals from raising alcohol-related concerns with their patients. More emphasis upon how to deal with alcohol-related problems is needed in both undergraduate and postgraduate programs so that prospective and current health professionals are equipped with the knowledge, awareness and confidence in addressing alcohol-related issues as part of their daily professional practices [75].

Additionally, there is a need for greater awareness of the available alcohol-related treatments and the potential benefits of specialist support. We only identified six intervention studies which assessed professional practice change so that their potential for widespread use requires further testing. However, it appears that multifaceted interventions, tailored to suit both individual and group needs supported by written material, practice guidelines including screening tools and BIs, and experiential learning are most effective. Further evaluation of these interventions over the short- and long-term will be important to determine the extent of attitude and personal and professional behavior change to inform continuing professional development initiatives. Regular booster sessions may help to maintain the desired effect of any educational interventions [42]. There is also a need to use theory to underpin the design of educational interventions so that the mechanism of behavioral change is explicit.

The review has also highlighted areas for future research. Strand 1 of the review found that most studies focused on the “Assess” (*n* = 18) and “Advice” (*n* = 13) components of the 5-As behavioral counseling framework [27] with fewer studies examining the “Agree” (*n* = 9), “Assist” (*n* = 3) and “Arrange” (*n* = 9) components. Furthermore, none of the studies examining the effect of interventions upon professional activity reported their effect upon “Assist” and “Arrange” activities. Although a range of professional alcohol-related health promotion practices are currently being conducted, no study has examined all of the components of the 5-As behavioral counseling framework [27]. While future studies should continue to report health professionals’ practices in terms of initiating informal discussions about alcohol use with their patients, confidence in using screening tools, and providing general and specific advice, future studies should also investigate the ways in which health professionals assist their patients by providing written information, goal setting, counseling, specialist support, and referral to appropriate drug and alcohol counseling services if required. 

It is surprising that only a few studies have investigated the relationship between health professionals’ personal alcohol attitudes and behaviors, and their professional alcohol-related health promotion practices. Future studies should investigate the extent to which health professionals’ own health attitudes, beliefs and practices influence their professional alcohol-related practices. Valid standardized measures should be used to supplement self-report data provided by participants to enable comparisons across studies and the development of firm conclusions about current trends. 

## 5. Conclusions

While there is some evidence of alcohol-related health promotion within the clinical practice of health professionals, the evidence is limited and suggests that there is a need for more health promotion activity if the harmful use of alcohol and its health and social consequences are to be addressed effectively. Additionally, the review highlights the potential relationship between the personal alcohol use and attitudes of health professionals and their professional alcohol-related health promotion activities indicating both the need for further research and supportive alcohol-related initiatives focused upon the personal lives of practicing health professionals. Further well designed research is also needed to provide a sound evidence base to inform the development of alcohol-related health promotion activities of health professionals. This may include the testing of educational interventions to extend the use of the 5-As behavioral counseling framework or similar comprehensive protocols addressing the hazardous alcohol use of patients within the daily clinical practice of health professionals as part of public health. 
